# Dr George L. Bakris, 1952-2024

**DOI:** 10.1016/j.jscai.2024.102279

**Published:** 2024-08-16

**Authors:** Michael H. Davidson

**Affiliations:** Lipid Clinic, The University of Chicago Pritzker School of Medicine, Chicago, Illinois

George L. Bakris, MD, a veritable giant in the field of hypertension and the prevention of cardiovascular and kidney disease, died on his 72nd birthday, June 15, 2024, after a courageous battle against anaplastic thyroid cancer. Typical of George, he worked almost to the end, seeing patients, lecturing, and overseeing clinical trials. He published more than 800 peer-reviewed manuscripts in a career that spanned nearly 4 decades, many in leading medical journals, including his most recent *The New England Journal of Medicine* publication this month on a large clinical trial demonstrating the effects of semaglutide on chronic kidney disease in patients with type 2 diabetes. Clinical trials, caring for patients, and lecturing all over the world were his passions, and his impact on patient care will be known for generations to come.

**Figure 1:**
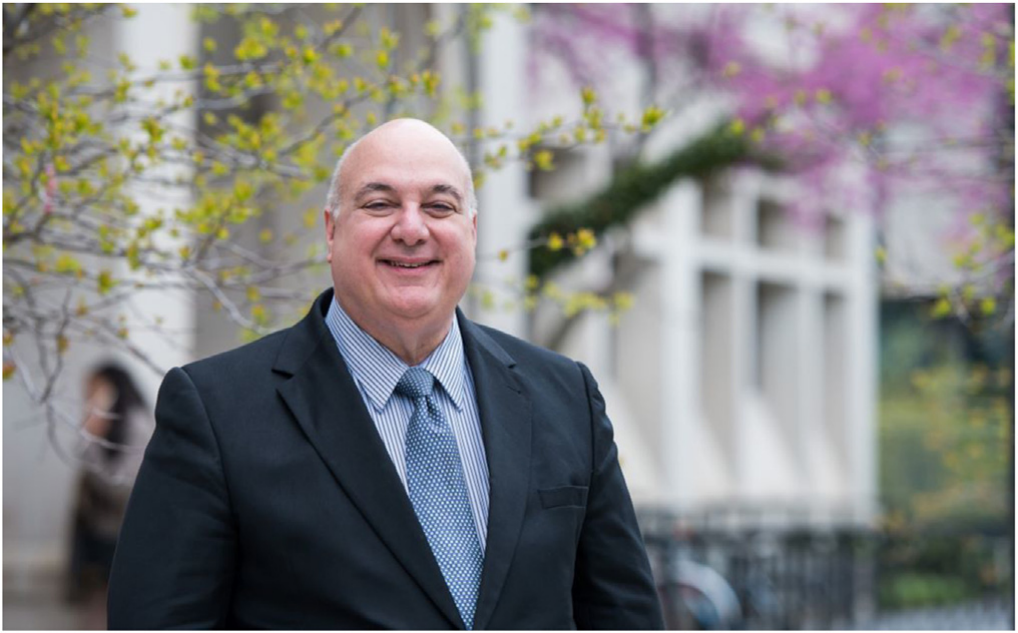
**George L. Bakris, MD, pictured in 2017.** Reproduced with permission from the photographer (Jean Lachat).

George was raised as an only child by older Greek immigrants in South Bend, Indiana. Dr Bakris graduated from Indiana University Bloomington in 1974 with a bachelor’s degree in biology and psychology. He pursued medical studies at the Rosalind Franklin Chicago Medical School, graduating with distinction in 1981. He trained as a nephrologist at The University of Chicago and started his academic career with special expertise in hypertension. After his humble Midwest origins, George became intimately involved in the “golden age” of hypertension clinical trials at Rush University Medical Center where he was working with Dr Henry R. Black and ultimately became vice chair of the department of preventive medicine before moving to The University of Chicago (Figure 1) as director of the Comprehensive Hypertension Center in 2006.

George’s impact on the contemporary management of hypertension cannot be overstated. Early in his career, he advocated for lower-dose combination therapy, angiotensin-converting enzyme inhibitors, and angiotensin II receptor blockers for reducing kidney disease progression, and over the years conducted many of the pivotal trials establishing the benefits of SGLT2 inhibition, GLP-1 agonism, and finerenone for renal dysfunction. The breadth of his research encompassed all aspects of hypertension care, including lifestyle change to novel pharmacotherapeutic agents and combination therapy to catheter-based renal denervation (RDN). With regard to the latter, George was one of the early champions for RDN, a promising technology that finally gained U.S. Food and Drug Administration approval less than a year ago and nearly 1 decade after the publication of the SYMPLICITY HTN-3 trial in *The New England Journal of Medicine*, a pivotal study for which he served as the national co-principal investigator and senior author. The refinement of RDN and exploration of its effects beyond the management of resistant hypertension served as important touchpoints with the global interventional cardiology community for the last 15 years of his career.

I was blessed to know George as a colleague working together in the clinic at The University of Chicago. He loved patient care, and in return, his patients worshiped him. Each year he mentored a new hypertension fellow, many from his native Greece. George, with his brilliant mind, gave lectures that were unmatched for teaching health care professionals—both the art and the science of clinical care. To those who knew George, we will miss most his good nature, his willingness to help others, his amazing ability to speak authoritatively about almost any subject, and his rare ability to make others feel good about themselves in his presence. True to his humble beginnings, George’s obituary in the local Indiana newspaper wrote that “He is survived by his loving wife and best friend of over 40 years (Demetria), children (Athena [Matt] and Louis [Despina]), and grandchildren. George leaves behind a legacy that extends beyond his family and local community, and spans the globe.”

